# Selective collection of iron-rich dust particles by natural *Trichodesmium* colonies

**DOI:** 10.1038/s41396-019-0505-x

**Published:** 2019-09-24

**Authors:** Nivi Kessler, Rachel Armoza-Zvuloni, Siyuan Wang, Subhajit Basu, Peter K. Weber, Rhona K. Stuart, Yeala Shaked

**Affiliations:** 1 The Fredy and Nadine Herrmann Institute of Earth Sciences, Edmond J. Safra Campus, Givat Ram, Hebrew University of Jerusalem, Jerusalem, Israel; 2 Department of Plant and Environmental Sciences, The Alexander Silberman Institute of Life Sciences, Edmond J. Safra Campus, Givat Ram, Hebrew University of Jerusalem, Jerusalem, Israel; 3 Interuniversity Institute for Marine Sciences, Eilat, Israel; 4 Physical and Life Sciences, Lawrence Livermore National Laboratory, Livermore, CA USA; 5 Present Address: The Dead Sea-Arava Science Center (DSASC), Hevel Eilot, Israel

**Keywords:** Biogeochemistry, Biogeochemistry, Water microbiology, Environmental sciences

## Abstract

Dust is an important iron (Fe) source to the ocean, but its utilization by phytoplankton is constrained by rapid sinking and slow dissolution dust-bound iron (dust-Fe). Colonies of the globally important cyanobacterium, *Trichodesmium*, overcome these constraints by efficient dust capturing and active dust-Fe dissolution. In this study we examined the ability of *Trichodesmium* colonies to maximize their Fe supply from dust by selectively collecting Fe-rich particles. Testing for selectivity in particle collection, we supplied ~600 individual colonies, collected on multiple days from the Gulf of Aqaba, with natural dust and silica minerals that were either cleaned of or coated with Fe. Using a stereoscope, we counted the number of particles retained by each colony shortly after addition and following 24 h incubation with particles, and documented translocation of particles to the colony core. We observed a strong preference for Fe-rich particles over Fe-free particles in all tested parameters. Moreover, some colonies discarded the Fe-free particles they initially collected. The preferred collection of Fe-rich particles and disposal of Fe-free particles suggest that *Trichodesmium* can sense Fe and selectively choose Fe-rich dust particles. This ability assists *Trichodesmium* obtain Fe from dust and facilitate its growth and subsequent contribution to nutrient cycling and productivity in the ocean.

## Introduction

In large parts of the oceans, short supply and restricted availability of iron (Fe) limit phytoplankton growth [1–3]. Wind driven desert dust is a major source of new Fe to remote oceanic regions [2, 4, 5], supplying Fe-bearing minerals to Fe-depleted surface water [6–8]. Mineral-Fe is not directly available to most phytoplankton, which can only internalize soluble Fe [9]. Hence, dissolution of dust-bound Fe is a prerequisite for its utilization by phytoplankton [10, 11]. Desert dust contains a complex matrix of minerals, varying in structure, size, and Fe content. Some dust minerals, such as Fe-oxides and hydroxides, are composed mostly of Fe (50–80%), while other dust components are relatively poor in Fe [12, 13]. Fe release rates from dust minerals are influenced by multiple factors in addition to their Fe content, such as size, mineral structure, weathering degree, and reactions that occurred in the atmosphere during transport and in the ocean microlayer upon deposition [14]. The residence time of dust minerals in the ocean surface can vary from days to months [15, 16], during which a variety of processes (biological, photochemical, and chemical) can further transform the minerals and influence their bioavailability [17].

*Trichodesmium* is a successful, filamentous, colony-forming cyanobacterium residing in oligotrophic tropical and subtropical oceans. *Trichodesmium*, a globally important diazotroph (N_2_-fixer), contribute annually 60–80 Tg of new N, almost 40% of global marine N_2_ fixation [18]. Diazotrophy involves Fe-rich apparatus and requires a high inner quota of Fe [19]. *Trichodesmium* can exist in the water column as single filaments (trichomes) and as large colonies (~1 mm in diameter) that are visible to the naked eye [20]. Colonies consist of aggregates of several to several hundred trichomes and form fusiform colonies when aligned in parallel (called “tufts”), or spherical colonies when aligned radially (called “puffs”). Various species of *Trichodesmium* have been described based on the morphology and structure of colonies [21] and recently, using genetic tools, were grouped to two clades that probably inhabit different ecological niches [22].

The low surface area to volume ratio of large *Trichodesmium* colonies imposes a strong limitation on dissolved Fe acquisition [23]. Hence, to sustain its high Fe demands *Trichodesmium* relies on Fe supply from particles, such as air-borne dust deposited on the ocean surface [24–27]. *Trichodesmium* holds various physiological adaptations that enable it to physically interact and retain dust particles. It can regulate its buoyancy and tends to form dense blooms right at the ocean surface, where air-borne dust settles [18]. In addition, the large surface area and intricate morphology of *Trichodesmium* colonies enable effective capture and retention of dust particles compared with single-cell phytoplankton [28]. In addition to physical associations with particles, natural tufts and puff-shaped colonies were shown to actively move dust particles [24] and shuttle dust from the periphery to colony core in a coordinated movement of the trichomes [25].

Intrigued by *Trichodesmium*’s unique ability to actively collect dust particles, we set out to explore whether *Trichodesmium* colonies select the particles they capture and maintain in their core. An ability to sense the presence or absence of Fe on/in particles will enable *Trichodesmium* colonies to optimize their particle collection activity, maximize Fe supply, and minimize possible costs of carrying non-nutritional particles. Testing for selectivity in particle collection, we conducted detailed microscopic observations on ~600 natural *Trichodesmium* colonies from the Gulf of Aqaba that were incubated for 24 h with natural dust and silica minerals that were either cleaned of or coated with Fe. Since Fe is present in a wide range of forms and particle sizes in seawater, we also conducted a ^57^Fe tracer experiment, incubating natural colonies with submicron ^57^Fe hematite, to test whether this small and less soluble form of Fe could also be sensed and centered. To examine the particle size range that could be actively centered at the submicron scale, we then imaged the colonies using NanoSIMS. Last, we studied cultured *Trichodemium erythraeum* (IMS101), finding that the morphological shift from single filaments to colonies was critical for their associations with dust.

## Methods

### Examination of selectivity in particle collection by natural Puff-shaped *Trichodemium* colonies

#### Colony collection and preparation

*Trichodesmium* colonies from the Gulf of Aqaba at the Northern Red Sea (29.56°N, 34.95°E) were collected with 200 µm plankton net subjected to ambient currents. The net was deployed from the pier of the Interuniversity Institute for Marine Sciences in Eilat (IUI) for short time intervals (2–3 h). Puff-shaped colonies that exhibited 3–4 different morphotypes (Fig. S1) were quickly handpicked from the net-concentrate under a stereoscope, using plastic droppers and suspended in a petri dish with filtered seawater (FSW). This collection scheme applies only a minimal stress on these delicate colonies, so that they remain integral over time (stressed colonies often tend to open up to individual filaments) [29]. Particle-free, integral, and well-formed puff-shaped colonies were selected and rinsed by three transfers to fresh FSW and then placed in separate wells of a round-bottom 96 well plate and allowed to acclimate for 1 h in 300 µl FSW prior to particle addition. Colonies were distributed equally and randomly between treatments, typically as 8–12 colonies per treatment.

#### Particle preparation and types

Fe-free and Fe-rich particle batches were prepared as detailed below and stored in double-distilled water (DDW). Essentially, Fe was removed from dust and silica particles (quartz and diatom frustules) via strong acid extraction, and then aliquots of these acid-cleaned particles were coated with a thin layer of Fe-oxides. Prior to each experiment diluted particle suspensions were prepared in FSW.

*Source material*—suspended dust was collected at the IUI, Eilat, a site receiving dust from Sahara desert, Arabian peninsula, and local sources [30–32]. The dust was sieved through a 200 µm mesh to remove large particles and fibers and was washed in FSW prior to incubations to minimize metal toxicity. Large quartz grains were collected from the IUI beach, examined by microscopy, and ground with a mortar and pestle. Diatom frustules were purchased as Diatomaceous Earth.

*Fe removal and addition*—dust and silicate particles were soaked in 32% hydrochloride acid for 2 weeks to extract all Fe from the minerals [30]. While silicates are resistant to acid, many of the dust minerals, such as carbonates, were digested by the acid, and hence we regard it as Fe-free modified dust. The acid-treated particles were thoroughly washed in DDW to remove any acid residue. Aliquots of these particles were coated with Fe-oxide following a procedure from [33]. Briefly, particles were added to 0.2 M Fe solution at pH of 2 (prepared from FeCl_3_). The mixture was slowly titrated with NaOH to a pH of 4.5–5.0 and then shaken vigorously for 24 h. The Fe-coated particles were repeatedly rinsed in DDW to remove free Fe. Washed particles were heated overnight at 90 °C to stabilize the Fe-oxides (and minimize their adhesiveness). The Fe-coating was apparent by color change, as Fe-coated particles appeared more reddish to the naked eye. Analysis of coated particles with Scanning Electron Microscope with Energy Dispersive Spectroscopy (SEM–EDS), confirmed coating by Fe (Fig. S2).

Considering the high Fe concentration in desert dust [34] and Fe-coated particles and the slow dissolution of these mineral phases in seawater, in the absence [35] and presence of *Trichodesmium* [29], we expect only moderate changes in the Fe content of these particles during our 24 h incubation.

#### Experimental determination of colony–particle interactions

Freshly collected, particle-free colonies were incubated with particles for 24 h at 25 °C and ~80 μE m^−2^ sec^−1^. Particles were added by pipetting a particle-seawater suspension into each well without mixing, to a final suspension density of 5 mg L^−1^. This particle suspension density was sufficient for rigorous observable interactions, for which particles abundance are not limiting. Colony-particle interactions were characterized by three measured parameters: short-term interaction (ST, tested 15 min after particle addition), centering (tested after 1.5 h), and long-term interaction (LT, tested after 24 h), as exemplified in Table 1. Each parameter was assigned a score: either no interaction (−), mild interaction (+), or strong interaction (++), as described in Table 1. Scoring was assigned by a trained experimentalist (same individual in all incubations), based on careful visual examination with a stereoscope. Verification and cross-checking of the scoring was conducted on images taken prior to (*T*_0_) and following particle addition (at 15 min, and 24 h, Fig. S3).

**Table 1:** Characterization scheme of particle collection by natural *Trichodesmium* colonies

#### Data analysis

The dependence between interaction scores and particle types was analyzed in contingency tables, using IBM SPSS software. Significance of deviation from the null hypothesis (=interaction scores are independent of particle type) was determined by Fisher’s exact test (this test was chosen since it is valid for all sample sizes). Association strength was assessed using Cramer’s V measure of association. To ensure that seasonal changes are not skewing the comparison between different particle types, caution was taken to compare only particle batches that were tested with the same number of colonies in each experiment day; silicates and dust did not have matching sample sizes in all experiment days and are hence were not compared.

### ^57^Fe tracer experiment and NanoSIMS and SEM–EDS analysis

We also examined if natural colonies collect submicron ^57^Fe-enriched hematite (Isoflex, 95% atom), by incubating them with 100 nM ^57^Fe-hematite in FSW for 24 h, at 25°C and 80 μE m^−2^ sec^−1^. To control for abiotic effects, some colonies were killed with 2% glutaraldehyde (1 h soak), washed with FSW, and incubated in parallel to live colonies. After incubation, colonies were placed on polycarbonate filters, washed with distilled water to remove salts, air-dried, and then coated with 15–20 nm of gold using a Hummer sputter coater (Anatech) to create a conductive surface. Colonies were then mapped with an Inspect F SEM (FEI) to identify target areas, which were analyzed using high-spatial resolution secondary ion mass spectrometry (SIMS) with a NanoSIMS 50 (CAMECA) at Lawrence Livermore National Laboratory equipped with a Hyperion II inductively coupled RF plasma ion source (Oregon Physics). Target areas were sputtered to a depth of ∼60 nm with a 600 pA O^−^ beam to reach sputtering equilibrium [36] before analysis with a focused 100–120 pA O^−^ beam scanned over a 50 μm × 50 µm square area with 512 × 512 pixels to generate secondary ions. Magnetic peak switching was used to detect secondary ions in two groups ([^40^Ca^+^, ^56^Fe^+^] and [^12^C, ^39^K^+^, ^31^P^+^, ^57^Fe^+^]) on electron multipliers in pulse counting mode (“combined analysis” mode). The metal ion peaks were identified using NBS610 glass (NIST). Each analysis area was scanned 5 times with 500 ns pixel^−1^ dwell times to collect serial secondary ion images for quantification of surface material. NanoSIMS ion image data were processed using L’IMAGE software (L.Nittler, Carnegie Institution of Washington) run on IDL (Harris Software). For each raster, quantitative ion images were generated for each of the masses.

### *Trichodesmium erythraeum* IMS101—culturing and interactions with dust

Strain IMS101 was grown at 25–26 °C, 12:12 h photoperiod at ~80 μE m^−2^ sec^−1^ in a modified YBCII medium with 20 μM EDTA and either low (50 nM) or high (400 nM) Fe [29]. Growth was monitored by in vivo florescence and by trichome counting in a Sedgewick-Rafter cell (Pyser-SGI, Kent, 157 UK) under bright-field Nikon Eclipse Ci-E microscope at ×10 magnification. Culture aliquots, which contained only single trichomes, were harvested at mid-exponential (13 days) and early-stationary (21 days) growth and incubated for 3 h with dust. The trichome-dust associations were visually examined at ×20–40 magnifications (enabling detection of ≥1 µm diameter particles). At least 2000 trichomes were examined for each treatment using a Sedgewick-Rafter cell. Colony formation was induced by incubating overnight concentrated (~tenfold) culture in nutrient poor FSW or YBCII with 50 nM Fe. Dust was mixed with the colonies for 3 h, and then individual colonies were transferred to 96 well-plate for stereoscopic examination of their associations with dust.

## Results

### Selective particle collection by natural *Trichodesmium* colonies—preference for Fe-rich over Fe-poor particles

During 2 months of high *Trichodesmium* abundance, we conducted 16 days of experiments in which we incubated ~600 individual natural colonies with natural dust and silica minerals that were either cleaned of or coated with Fe for 24 h (incubations outline in Table S1). In each experiment day, we randomly split the 30–60 freshly collected colonies to identical size groups (typically 8–12 colonies), added particles, observed, scored, and documented the three interaction parameters (see scoring criteria in Table 1). Each individual colony was assigned a score for each interaction parameter. Dust types were tested in all experiment days, while silicate particles were tested in only 8 of the days.

**Fig. 1:**
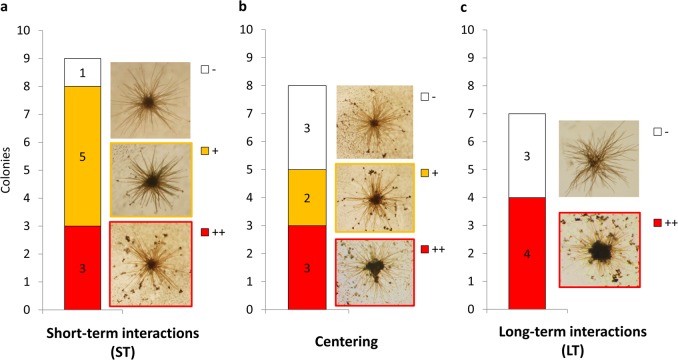
Interactions between natural *Trichodesmium* colonies and dust in a single experiment day. In the experiment conducted on March 8th 2016, 27 colonies were collected and divided to three groups, each incubated with one particle-type: natural dust, Fe-free dust, and Fe-coated dust. Interaction scores of the nine colonies incubated with natural dust are hereby presented (see Table 1 for parameter description and scoring criteria). Each bar represents the scores of colonies in one interaction parameter; red (++) = strong interaction, orange (+) = mild interaction, and white (−) = no interaction. Pictures show typical appearance of colonies that were assigned these scores. **a** Short-term interaction (ST) scores assigned to all examined colonies. **b** Centering scores assigned to the eight colonies that interacted with dust (i.e., positive ST). **c** Long-term interaction (LT) scores assigned to the seven colonies that remained integral following overnight incubation

To clarify the procedure and make the following graphs accessible, we first present the results of a single treatment (natural dust addition) from a single day (March 8th 2016), showing the cumulative scores of the colonies (Fig. 1). The first score, ST interaction with dust, reflects the number of particles that adhered on the colonies within 15 min from dust addition. Here, colonies readily interacted with dust and hence ST scores are mostly positive, as indicated by the orange (+) and red (++) colors (Fig. 1a). Only one colony was plotted as white (−) since it retained no particles (Fig. 1a). We then examined the degree of particle centering over 1.5 h duration in the eight colonies that initially interacted with dust. Since the centering score represents the fraction of the adhered particles that were moved toward the colony center, the “empty” colony could not be scored. We found that five colonies moved the dust to their core (and hence were scored positive), while in the other three colonies the dust was still scattered along the filaments (Fig. 1b). Finally, following an overnight incubation we determined the LT particle load on colonies that maintained their morphology. Here, of the seven colonies that remained integral, four kept accumulating particles and were heavily loaded with dust (labeled red), while three were free of particles (labeled white, Fig.1c).

#### Selective collection of Fe-rich particles—population level

**Fig. 2:**
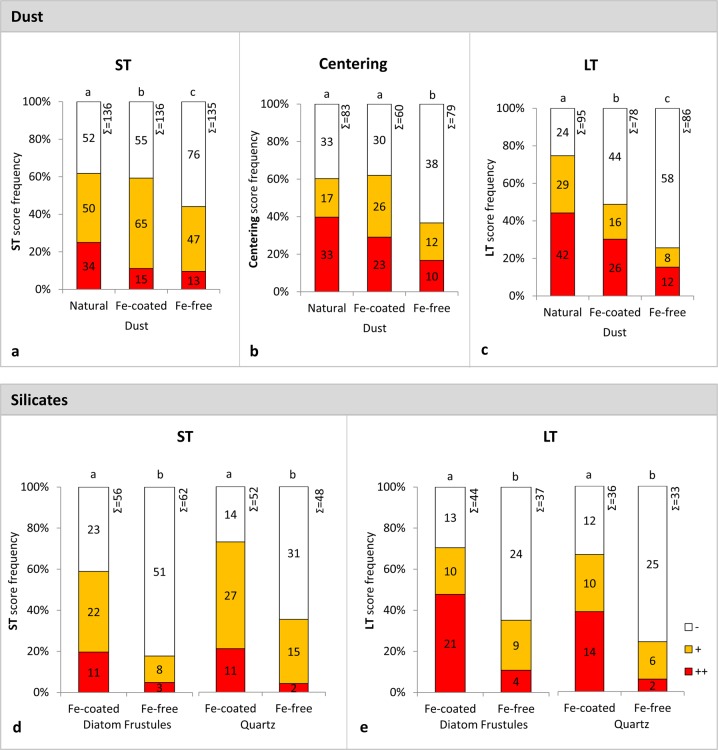
Interactions between natural *Trichodesmium* colonies and different particles complied from all 16 experiment days. Top panels (**a–c**) show incubations with dust types and bottom panels (**d**, **e**) incubations with silicates, grouped by interaction parameter: short-term (ST) interaction (**a**, **d**), centering (**b**), and long-term (LT) interaction (**c**, **e**). Data from all 16 days of experiments was pooled together. The numbers of colonies in each category appear on the bars and the overall colony numbers at top of the bars. Interaction strength is noted by colors where red (++) = strong interaction, orange (+) = mild interaction, and white (−) = no interaction. Bars show the frequency of scores (−, +, ++) for each category. Statistically significant differences between treatments were evaluated using a 2-sided Fisher’s exact test, *P*-value threshold of 0.05. Statistically different treatments, within the same parameter, are assigned different letters (above column). Data on centering of silicates are shown in Fig. S5 (statistical analysis not reliable since too few colonies interacted with Fe-free silicates)

We analyzed our data in several ways, starting by pooling all 16 experiment days together. We present the interaction with dust types (Fig. 2, top panel), separate from those with silicate particles (Fig. 2, bottom panel). Statistically significant preference for Fe-rich over Fe-poor particles was observed for all interaction parameters (see Table S2 for statistical information).

More colonies interacted initially (ST parameter) with natural and Fe-coated dust (~60% positive colonies) than with Fe-free dust (45% positive colonies, Fig. 2a). Similarly, more colonies shuttled natural and Fe-coated dust toward their colony core (centering parameter) than Fe-free dust (Fig. 2b). Lastly, strong preference for natural dust over all other dust types was seen in the overnight incubation (LT parameter), with 75, 50, and 25% of the colonies retaining natural, Fe-coated and Fe-free dust, respectively (Fig. 2c). Fe-free dust was pretreated in acid that removed other dust components in addition to Fe, such as carbonate minerals and other metal-oxides. Hence the lower collection, centering, and retention of these particles may not exclusively reflect sensing of Fe and selection of Fe-bearing minerals. However, the preferred interactions with Fe-coated dust over the original acid-washed dust (i.e., Fe-free; Fig. 2a–c), provides a direct indication for specific sensing of Fe and selection of Fe-containing particles.

We further verified *Trichodesmiu*m’s selection of Fe-containing particles using two silicate particles—diatom frustules and quartz. These particles are abundant in dust, were detected within natural colonies (Fig. S4), and are not modified by the strong acid required to leach out Fe. Despite their similar chemical composition, the smooth-surfaced quartz is highly distinct from the complex-surfaced diatom frustules, allowing us to test the effect of particle-surface features on the interaction with the colonies. The colonies showed a strong preference towards Fe-coated over Fe-free silicates in both ST and LT interactions (Fig. 2d, e). The initial (ST) preference for Fe-rich particles was more pronounced in silicates than in dust types, where 60% of the colonies interacted with the Fe-coated diatom frustules but only 20% interacted with Fe-free diatom frustules (Fig. 2d). In fact, the number of colonies that collected Fe-free silicates was too low to support a statistical comparison between particle types for the centering parameter (Fig. S5), and hence those data are not shown (see statistical analysis in Table S2). Particle surface morphology did not significantly affect the interaction parameters (Table S2), reaffirming that our data on *Trichodesmium*’s particle selection is not skewed by possible morphological changes that may have occurred during the acid treatment or Fe-coating of the particles. Combined, the incubations with dust-types and silicate particles indicate that *Trichodesmium* prefer and selectively collect and accumulate Fe-rich particles compared to Fe-free particles.

#### Selective collection of Fe-rich particles—temporal variations

**Fig. 3:**
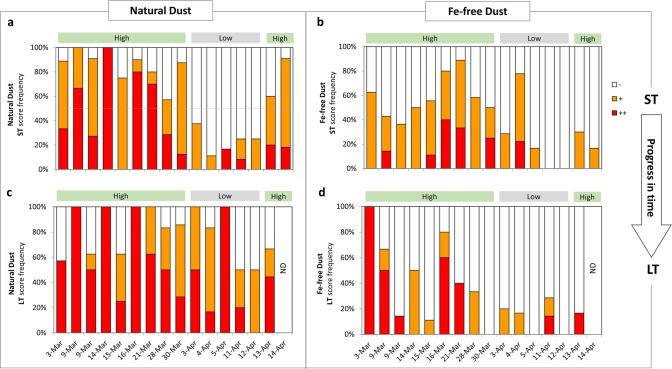
Seasonal changes in the interactions between natural *Trichodesmium* colonies and dust Daily scores of short-term (**a**, **b**) and long-term (**c**, **d**) interactions with natural dust (**a**, **c**), and Fe-free dust (**b**, **d**). Experiment dates are labeled on the *x*-axis and bars show the interaction strength (−, +, ++) as frequency. Over time the initial tendency of the colonies to interact with particles gradually changed. Applying a cutoff of 50% (dashed line in a) for ST interactions with natural dust, we defined two modes of initial particles collection—low and high (gray and green horizontal bars). These gradual changes appeared also for Fe-free dust (**b**). During overnight incubations, colonies kept accumulating dust (**c**) and removing Fe-free dust (**d**). ND no data

Moving from accumulated to single day data, we find that the colonies gradually changed their tendency to interact with particles during the season, as seen by the daily ST interaction scores (Fig. 3a, b). Based on ST interactions with natural dust we define two modes of initial particles collection—low and high (gray and green horizontal bars in Fig. 3). The cutoff was set to 50%, where in “low” days only 23 ± 10% of the colonies had positive ST scores (+/++), while in “high” days 84 ± 14% of the colonies had positive ST scores (Fig. 3a). A shift from “high” to “low” and then back to “high” was seen in both natural dust and Fe-free dust, but the later had noticeably lower daily ST interactions during the “high” days (52 ± 21% positive ST; Fig. 3b). A similar pattern was also observed for Fe-coated dust (Fig. S6). Interestingly, despite seasonal changes in ST interactions (Fig. 3a, b), the overnight interactions (LT) were consistent throughout the season; strong for natural dust (Fig. 3c) and weak for Fe-free dust (Fig. 3d). The increase with time in the interaction strength with natural dust (Fig. 3b is more “colored” than Fig. 3a) indicates that even colonies that did not interact initially ended up accumulating particles with time. On the other hand, for Fe-free dust, the strength of interaction declined during the overnight incubation (Fig. 3d is less “colored” than Fig. 3c). The drop in LT scores compared to ST scores that occurred in most of the experiment days suggests that colonies that initially interacted with Fe-free dust, discarded it during the overnight incubation.

#### Selective disposal of Fe-free particles

**Fig. 4:**
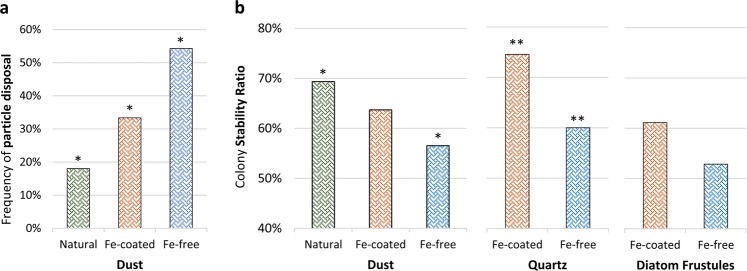
Selective particle removal (**a**) and particle effect on colony stability (**b**). **a** Frequency of individual colonies that, during an overnight incubation, removed the particles they initially collected. Fe-free particles were preferably removed over dust. **b** Fraction of colonies that remained integral throughout the incubation (defined as stability ratio). Colonies incubated with Fe-rich particles were more likely to remain integral compared to those incubated with Fe-free particles. Asterisk indicates statistically significant differences between the treatments, as evaluated using a 2-sided Fisher’s exact test, *P*-value threshold of 0.05

To further investigate the intriguing particle disposal behavior that emerged from the seasonal data, we compared between the long- and short-term interaction scores of each individual colony. A colony was defined as a “particle disposer” if its LT score was lower than its ST score. About 20% of the colonies that remained integral during incubation met this criterion, implying that they disposed of initially collected particles (data not shown). The percentage of colonies that discarded particles during incubation was higher for Fe-free than for Fe-rich particles (Fig. 4a). In fact, most colonies (55%) that interacted initially with Fe-free dust ended up discarding it within 24 h. In contrast only 18% and ~33% of the colonies discarded natural and Fe-coated dust, respectively. These intriguing findings suggest once more that *Trichodesmium* can sense the presence of Fe on/within particles. Moreover, the ability to discard Fe-free particles allows *Trichodesmium* to fine-tune its particle selection behavior in addition to favorably collecting Fe-rich particles.

#### Fe-rich particles contribute to colony stability

The tendency of a colony to retain its morphology or split to single filaments, during the 24 h incubation with particles, was also influenced by the presence of Fe. We define the percent of integral colonies at the end the incubation as a stability ratio, which ranged between 53 and 75% for different particle types (Fig. 4b). Stability ratios of colonies incubated with all Fe-rich particles, including natural dust, were higher than those incubated with Fe-free particles (Fig. 4b), indicating that Fe-bearing particles were beneficial for maintaining the colony morphology. Fe-rich minerals may have assisted *Trichodesmium* to remain as colonies via several pathways. Supply of iron from the minerals may have contributed to the colony fitness. Alternatively, the particles themselves served as a glue that kept the filaments together.

### Submicron Fe-rich particle collection by natural colonies

**Fig. 5:**
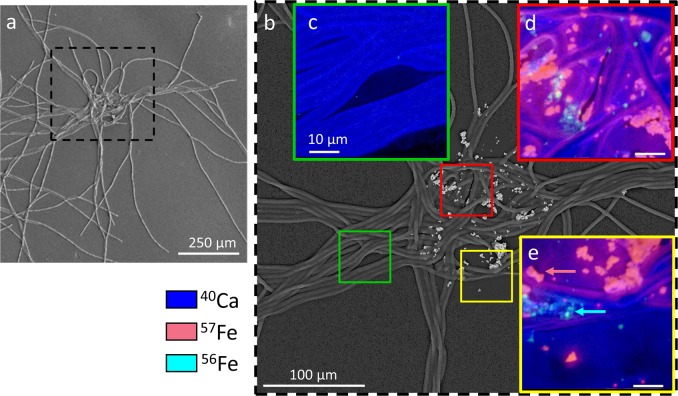
Representative image of natural colony incubated with isotopically labeled ^57^Fe-Hematite and analyzed using NanoSIMS. Left is a scanning electron microscopy image (**a**). Background image is a backscatter electron diffraction image (**b**), and inlaid images are 50 × 50 µm^2^ merged secondary ion images of surface distributions of ^57^Fe (pink), ^56^Fe (cyan), and ^40^Ca (blue) taken at different areas of the colony (**c**–**e**). In the colony periphery (**c**), the ^40^Ca-rich trichomes appear without any particles. The colony core (**d**, **e**) contains both the added ^57^Fe-Hematite particles (example indicated with a pink arrow), alongside ^56^Fe-rich particles collected by the colony from seawater before our incubations (cyan arrow). Figure S7 presents these and other NanoSIMS data as individual ion images

To extend our findings to additional types and sizes of Fe particles, we conducted a ^57^Fe tracer experiment with natural colonies. Hematite solubility in oxygenated seawater at natural pH is extremely low, and we questioned whether *Trichodesmium* would sense and select this highly stable Fe phase. Submicron scale surface imaging using NanoSIMS was done on natural colonies which were incubated with ^57^Fe-hematite (prepared from the rare Fe isotope ^57^Fe rather than the common isotope ^56^Fe), as well as control colonies which were chemically killed before ^57^Fe-hematite was added. Along with the ability to distinguish the added ^57^Fe from the more abundant ^56^Fe, the high resolution of the NanoSIMS enabled us to study the associations between natural colonies and submicron Fe-rich particles, which are not visible with stereoscope, and to conduct high-resolution imaging of different regions of the colonies. By generating detailed maps of different elements (Figs. 5c–e, S7, and single ion images—Fig. S8) we were able to clearly image the surface of the colonies (^40^Ca colored blue), the added ^57^Fe-hematite (^57^Fe colored pink), and the natural ^56^Fe-rich particles that the colonies collected from seawater before the colonies were harvested (^56^Fe colored cyan).

The added ^57^Fe-hematite was selected and retained in the colony core during incubation, indicating that it was sensed by the colonies despite its low solubility (Figs. 5, S7). Particles ranged from submicron (<400 nm) to micron-scale aggregates. In the colony core we also detected submicron (<400 nm) to 6 µm ^56^Fe-rich particles, which were collected in situ from seawater before our incubation (Figs. 5, S7, S8). The NanoSIMS data show a gradient of all Fe from high levels in and around the core toward low levels at the colony periphery, which was free of Fe-rich particles (Fig. 5). The sharp contrast between the particle-loaded core and the clean periphery provides further evidence for active transfer of particles. This active transfer of submicron particles occurred during incubation (as seen by ^57^Fe maps) and in situ (as seen by ^56^Fe maps). It is unclear from these data if the low-level ^57^Fe in the transition reflects nanometer-scale particles or solubilized or complexed Fe. The control chemically killed colonies retained some ^57^Fe-hematite but did not center it (Fig. S7). These findings adds to previous in situ imaging studies that either examined the trichome’s Fe content by synchrotron but found no particles [37, 38], or analyzed minerals associated with colonies with SEM–EDS [28], but lacked the high surface sensitivity and isotope tracer ability of the NanoSIMS.

### Do cultured *Trichodesmium erythraeum* (IMS101) interact with dust?

The data presented so far was obtained using freshly collected colonies from the Gulf of Aqaba, whose ecophysiology (such as nutrient status and growth phase) is poorly defined. Despite multiple attempts over several years, we were unable to culture any of the colony-forming natural strains. Hence, we chose to examine how Fe-limitation and growth phase affect *Trichodesmium*–dust interactions using strain IMS101. This strain typically grows as single filaments and can form colonies at conditions of stress [39]. We briefly present our findings as many laboratories use this strain for studying dust-nutrient availability [26, 40], but note that since IMS101 had a distinct behavior compared with natural colonies, extra care should be taken when extrapolating the laboratory-based observations to the ocean.

#### Interactions with dust occur upon morphological shift from single filaments to colonies

IMS101 did not form any associations with dust as long as it was growing as single trichomes (Table S3). Even Fe-limited and “old” (late exponential) cultures, conditions hypothesized as favorable for dust adhesion due to elevated production of extracellular polysaccharides [41], had low associations with particles (<2% of the trichomes; Table S3, Fig. S9).

Single IMS101 trichomes can aggregate and form colonies by increasing the attachment forces between the trichomes [39]. Speculating that colony forming IMS101 will also interact with dust, we first characterized the conditions leading to colony formation. To induce colony formation, low and high-Fe grown cultures were concentrated by ~tenfold and incubated overnight in nutrient-poor FSW or fresh YBCII media. High-Fe grown cultures did not form any colonies regardless of incubation media or time (Table S3). Low-Fe grown cultures formed abundant colonies (~80 colonies mL^−1^; Fig. S10) within 16 h of incubation in FSW, but not in YBCII (Table S3). Hence, the main drivers for colony formation, in our study, were Fe-depletion and high density, in accord with previous reports [39]. The newly formed colonies readily interacted with dust (Table S3). Despite the efficient and rapid adsorption of dust on IMS101 colonies, we did not detect active movement of particles within 24 h.

## Discussion

**Fig. 6:**
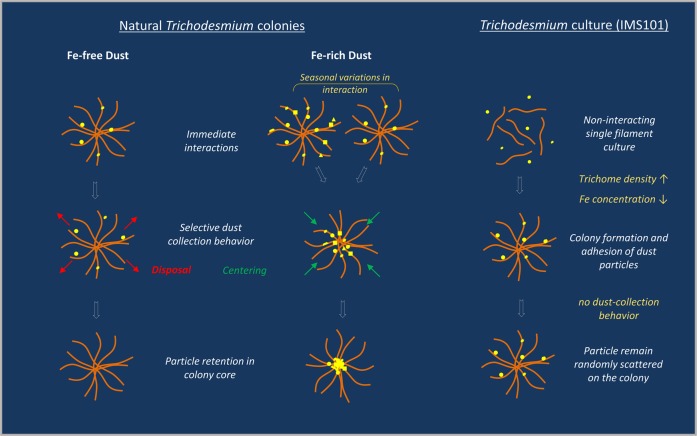
Schematic representation of *Trichodesmium*–dust interactions observed in this study and the role of Fe in these interactions. Fe-rich dust (middle) was preferably collected and concentrated, over Fe-free dust (left). Although the initial interactions with Fe-rich dust varied considerably over the season (middle-top), continuous dust collection during overnight incubation resulted in high particle retention (middle-bottom). Fe-free particles were not only collected to a lesser degree (top-left) but were also frequently removed from the colonies (middle-left), resulting in particle-free colonies (bottom-left). Experimenting with cultured strain IMS101 (right), did not yield any associations between dust and *Trichodesmium*, as long as the culture grew as single filaments (top-right). When colony formation was induced (at conditions of Fe limitation and high trichome density) the colonies readily interacted with dust (middle-right), but did not center it during an overnight incubation (bottom-right)

To assist data synthesis, we schematically illustrate the interactions with dust we observed for natural and cultured *Trichodesmium*, highlighting the role of Fe in these interactions (Fig. 6). For natural colonies, the presence of Fe within or on minerals was found to influence the ST colony–particle interactions, as significantly lower number of colonies interacted with Fe-free dust or Fe-free silicates, compared with dust or Fe-coated minerals (Figs. 2a, d, 6). Throughout the season, the number of colonies that formed positive ST interactions with dust gradually changed (Figs. 3a, 6). These seasonal changes may reflect shifts between *Trichodesmium* species or physiological changes that influence colony adhesiveness. After the initial interaction, colonies were found to further collect, center, and retain dust and Fe-rich particles in their cores, during an overnight incubation (Fig. 2b, c, e). The LT overnight continuous collection of dust and Fe-rich particles occurred throughout the season (Fig. 3c), even in days with weak initial interaction (Figs. 3a, 6). NanoSIMS analysis revealed that natural colonies contain Fe-rich particles, both those derived from the added ^57^Fe-hematite and natural sources (^56^Fe), that are too small to be detected by light microscopy (Fig. 5). Fe-rich particles were found almost exclusively in the colony core, and the peripheral areas were free of particles. These observations indicate that *Trichodesmium* actively collect and center Fe-particles from their natural environment and can interact with a wide range particle sizes, down to the submicron (Figs. 5, 6). However, the continuous particle collection was selective, and did not occur in all cases. We documented here for the first time that many colonies, in fact, discarded Fe-free particles (Fig. 4a). By disposing of Fe-free particles, these colonies “cleaned” their surfaces (Fig. 5c), and possibly got ready to collect new “nutritional” Fe-rich particles. Combined, our findings suggest that *Trichodesmium* can sense the presence of Fe on/in particles and that it coordinates the selective collection of dust and Fe-rich particles by a combination of physical interactions, centering and selective disposal of Fe-free particles (Fig. 6).

*Trichodesmium*–dust interactions of cultured IMS101 did not share much similarity with the natural puff-shaped colonies. As long as the culture was growing as single filaments, it did not form any interactions with dust, regardless of its growth phase or Fe-status (Table S3, Fig. 6). When colony formation was induced (at conditions of Fe limitation and high density) the colonies readily interacted with dust but did not center it during an overnight incubation (Fig. 6). Our findings of coupling between colony formation and dust adsorption complement those of Langlois et al. (2012) who grew IMS101 with dust and observed formation of colonies [26]. Together, these observations suggest that colony formation and physical interactions with dust are strongly related in strain IMS101 and may even be regulated by common pathways. However, these findings are not applicable to many coastal and open ocean ecosystems, where colonies prevail and even dominate the population under non-Fe limiting conditions. Basins receiving high Fe fluxes through sediment transport or aeolian dust deposition such as the Red Sea, North Atlantic, and Arabian Sea are often characterized by high abundance of colonies [24–28]. We hence propose that the ecological drivers and physiological mechanisms leading to colony formation, colony stability, and longevity and/or interactions with dust are complex and can vary among *Trichodesmium* species and within a single species over time. In particular, extra care should be taken when extrapolating IMS101 data related to metabolic functioning of colonies, drivers for colony formation, and associations with dust to oceanic settings, due to differences we and others documented [25, 42–44].

Back to the natural colonies, the selection of Fe-rich particles rather than Fe-free particles, suggests that *Trichodesmium* can sense that the minerals contain Fe. To the best of our knowledge, an Fe-sensing system in *Trichodesmium* was not described. Studies on sensing of Fe  by bacteria such as Pseudomonas report that Fe is sensed in its soluble form, as ions rather than minerals [42]. Hence, we propose that *Trichodesmium* relies on soluble Fe released from the mineral to discern if it contains Fe. The NanoSIMS images showing centering of ^57^Fe-hematite, a mineral often considered as insoluble in oxygenated high pH seawater, suggests that *Trichodesmium*’s sensing system is sensitive to minute levels of soluble Fe. The minerals we coated with Fe were also stabilized by heat, a process that diminished their solubility, but nonetheless selection of these particles occurred, signifying that low concentrations of soluble Fe were sensed. Although we focused here on Fe, another important limiting nutrient for *Trichodesmium* that may be delivered with dust is phosphorus (P). While removing Fe from dust with acid, P was also removed. Hence it is possible that the preference for natural dust over all other particle type examined (Fig. 2), may be due to the presence of  P (and possibly other elements). In such a scenario, *Trichodesmium* should be also able to sense P and selectively collect P-rich particles. Further study on chemical sensing in *Trichodesmium* is required to fully assess these suggestions.

Despite the postulated high sensitivity of *Trichodesmium’s* sensing system to low levels of soluble Fe, the low solubility of hematite and other Fe-minerals makes them poor suppliers of soluble Fe required for *Trichodesmium* growth. Since most of the Fe in dust is found as very stable mineral phases, the amount of Fe released from dust is rather minimal, as evident by frequent reports on Fe (and P) limitation in natural *Trichodesmium* populations in the North Atlantic that receive Saharan dust inputs [26, 27]. In recent years we documented a variety of biochemical pathways and physical mechanisms that assist *Trichodesmium* obtain some Fe from mineral sources. Dust packaging in the colony core is beneficial for uptake, since cell-particle proximity facilitate uptake of Fe that dissolves from dust prior to its loss by diffusion [25, 29]. Natural colonies can also enhance dissolution rates of dust-bound Fe [25] and Fe-oxyhyroxides such as ferrihyrite [29]. Siderophores, iron-complexing molecules synthesized by many bacteria (but not by *Trichodesmium*), were proposed to assist in dust-Fe dissolution [45, 46]. Lately we showed that *Trichodesmium* and its associated bacteria act together to increase availability of dust-bound iron, where bacteria promote dust dissolution by siderophore production and *Trichodesmium* provides dust and optimal physical settings for dissolution and uptake [44]. Our intriguing findings on selection of Fe-rich particles, adds to the arsenal of *Trichodesmium’s* unique adaptations for utilizing dust as an iron source.

Extending our findings to additional environments we make a distinction between environments with high and low particle loads. In high particle-load, coastal environments, the newly discovered ability of *Trichodesmium* to remove particles can be of physiological significance. Collection and centering of large numbers of particles may disturb buoyancy regulation, mask light penetration, and may expose the colonies to  heavy metals and toxic elements leaching from these particles [30–32]. These trade-offs can be offset if colonies can control the type and amount of retained particles and fine-tune particle nutritional value with their metabolic requirements. In low particle-load open-ocean, chemical sensing of particle composition and nature can assist in detecting and discarding invading bacteria or toxic particles. Returning to Fe, if *Trichodesmium* indeed sense dissolved Fe released from minerals, it may then select and retain fast Fe releasing or more soluble particles. Our findings from the Gulf of Aqaba, if shared by other *Trichodesmium* colonies in various ocean basins, can hence assist *Trichodesmium* optimize the collection and retention of dust to favor particles that can supply them with scare Fe (and P) and consequnetly contribute to nutrient cycling and productivity in the ocean.

## Supplementary information

Supplemental Material
