# Supplementary material for: Selective collection of iron-rich dust particles by natural Trichodesmium colonies
Source: ISME J. 2019 Sep 24;14(1):91–103. doi: 10.1038/s41396-019-0505-x (PMC6908701; doi:10.1038/s41396-019-0505-x)
Supplement: Supplementary file 1 — Supplemental Material [file 41396_2019_505_MOESM1_ESM.docx]

Supporting material for Manuscript:

Selective Collection of Iron-Rich Dust Particles by Natural *Trichodesmium* Colonies

By:

Nivi Kessler, Rachel Armoza-Zvuloni, Siyuan Wang, Subhajit Basu, Peter Weber, Rhona Stuart, and Yeala Shaked

Contains: 10 figures and 3 tables

| A | B |
| --- | --- |
| 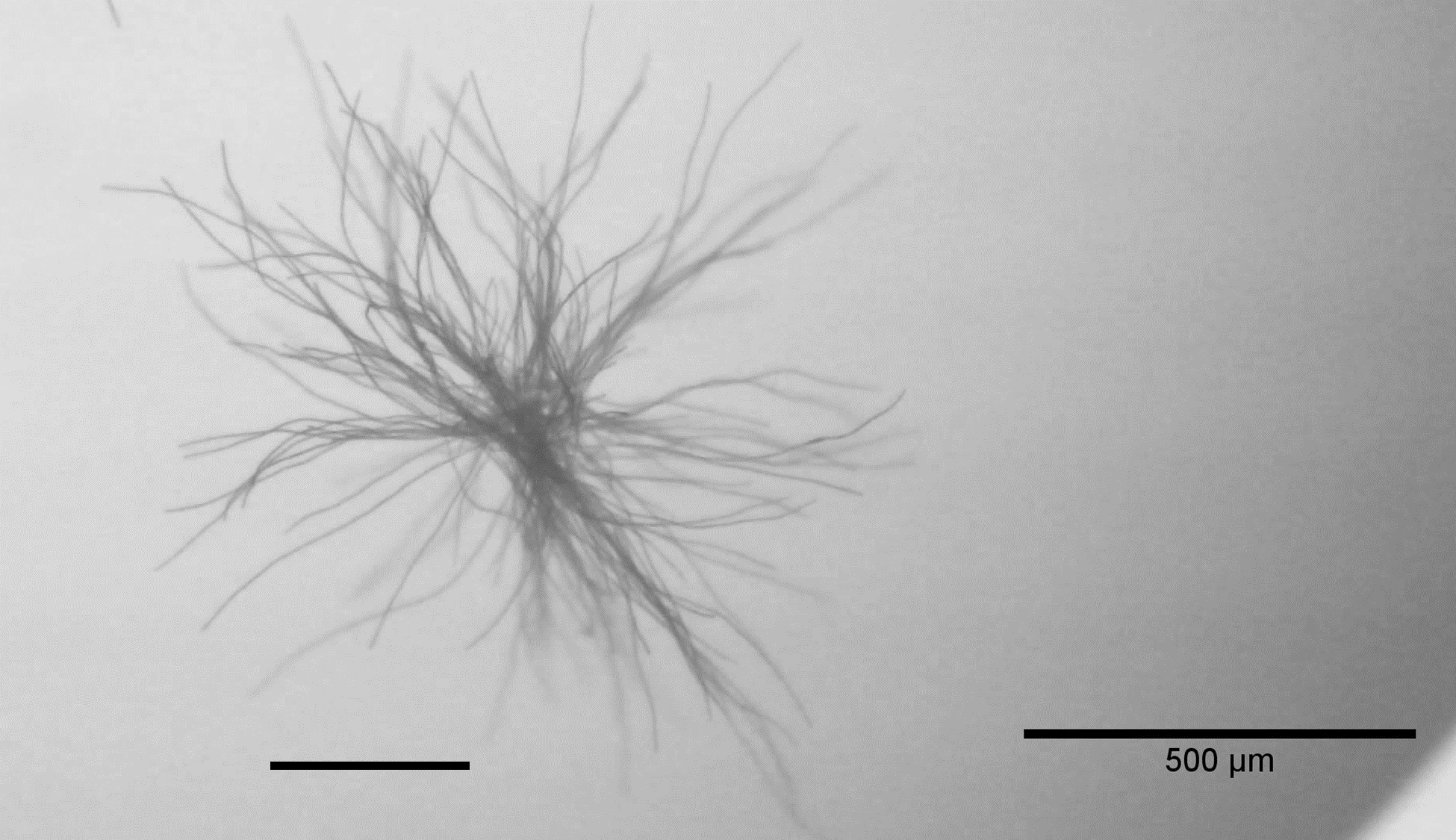 | 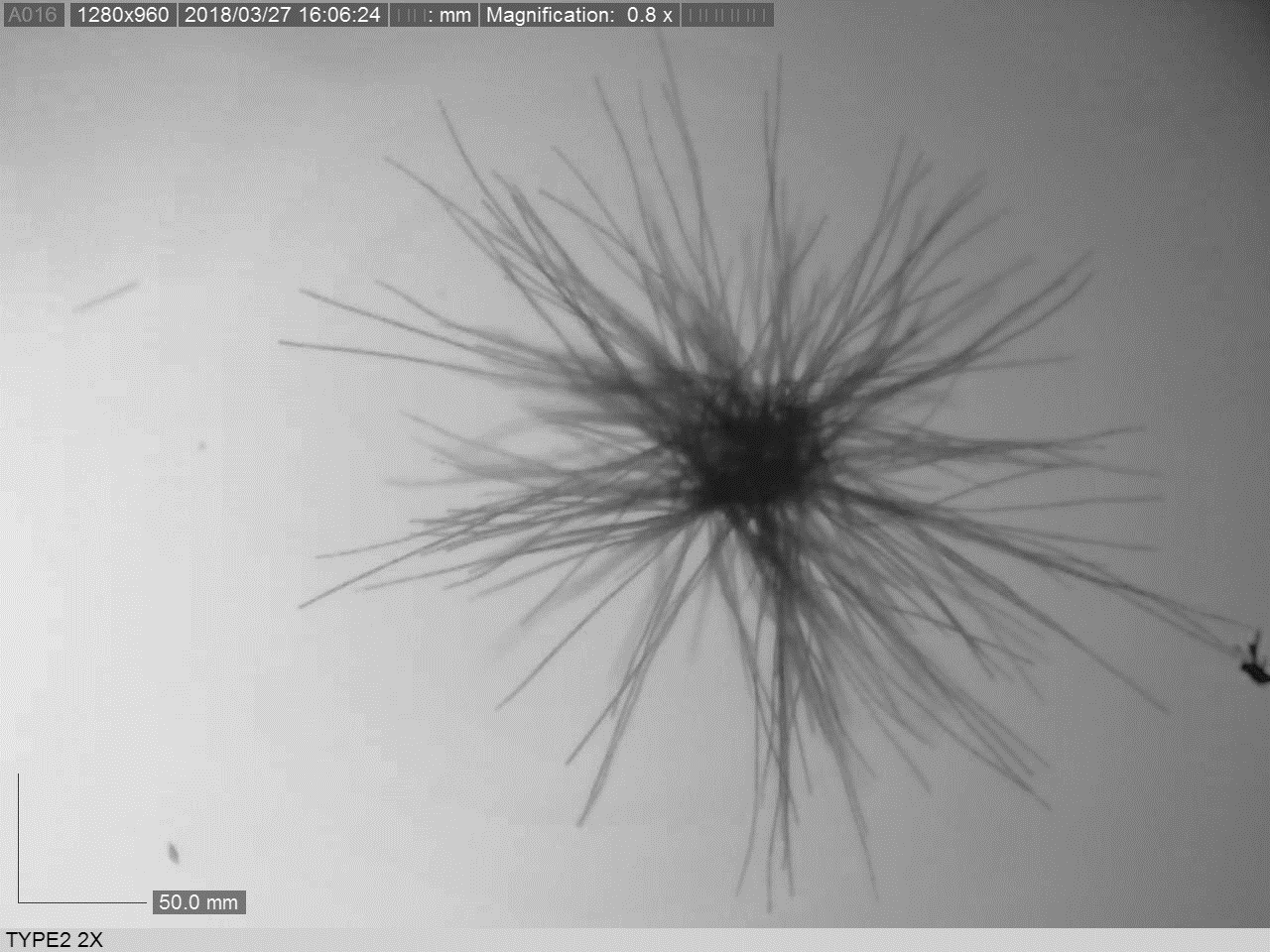 |
| C | D |
| 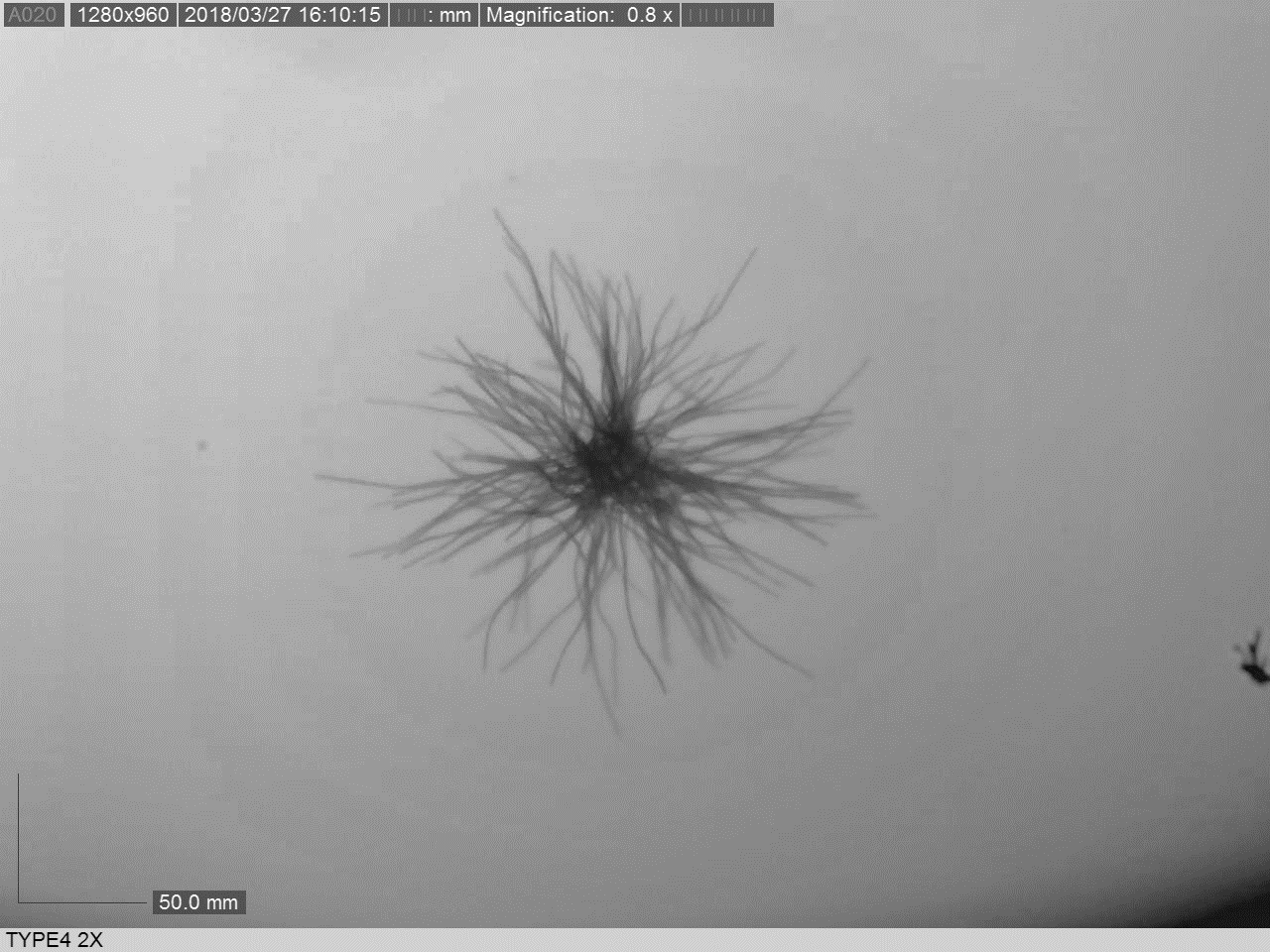 | 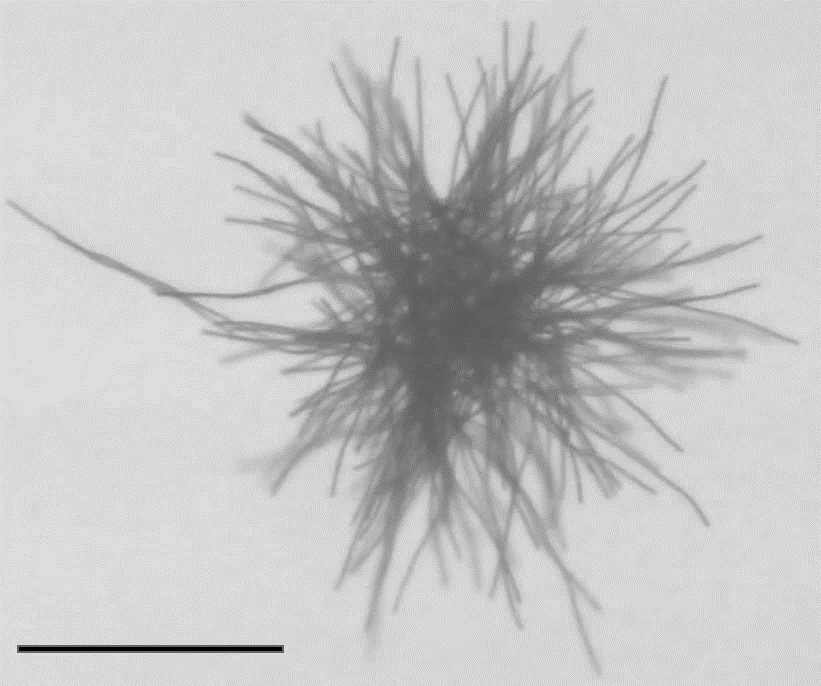 |
| **Figure S1: Colony morphotypes.** Natural *Trichodesmium* colonies, collected in the Gulf of Aqaba, were identified to have several typical morphotypes, which differ in trichome length and appearance and core density. Type I(A) - Long trichomes with a thin core; Type II (B) - Long trichomes with a dense core; Type III (C) - Short trichomes with a thin core; Type IV (D) - Short trichomes with a dense core. Scale bar- 500 µm. | |

| A | B |
| --- | --- |
| 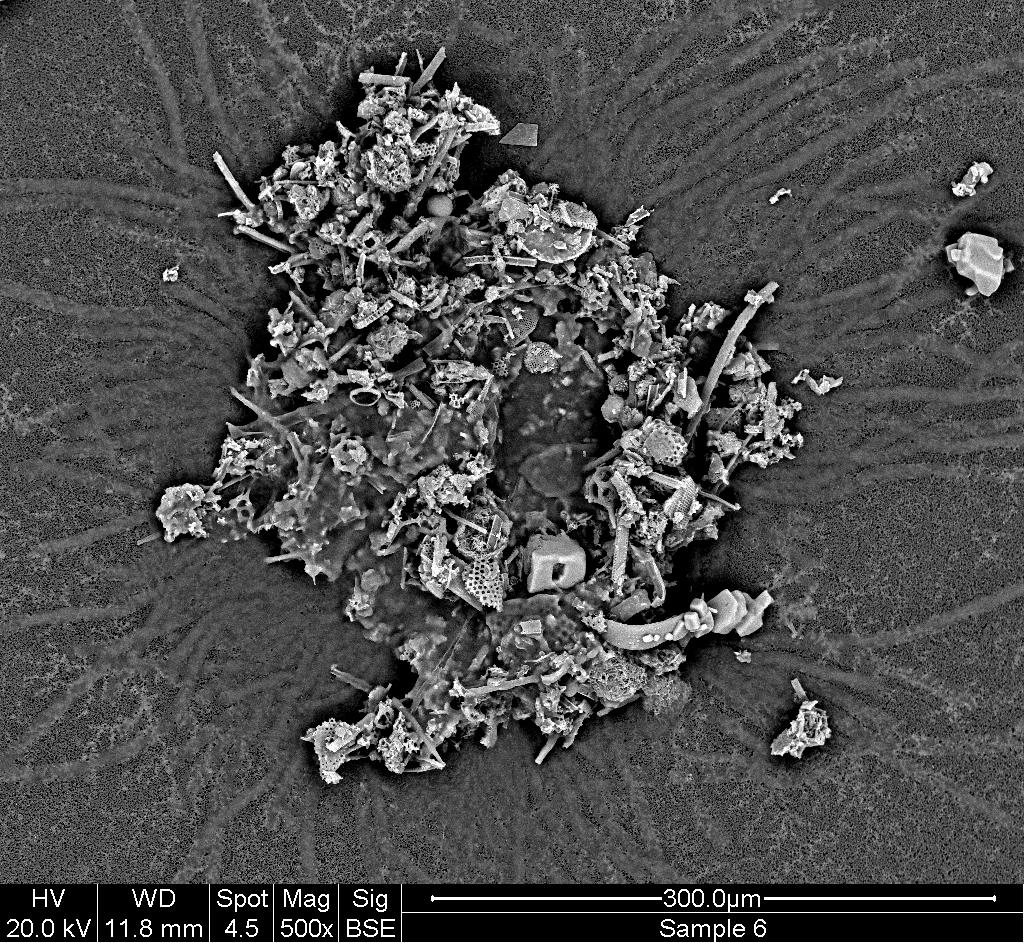 | 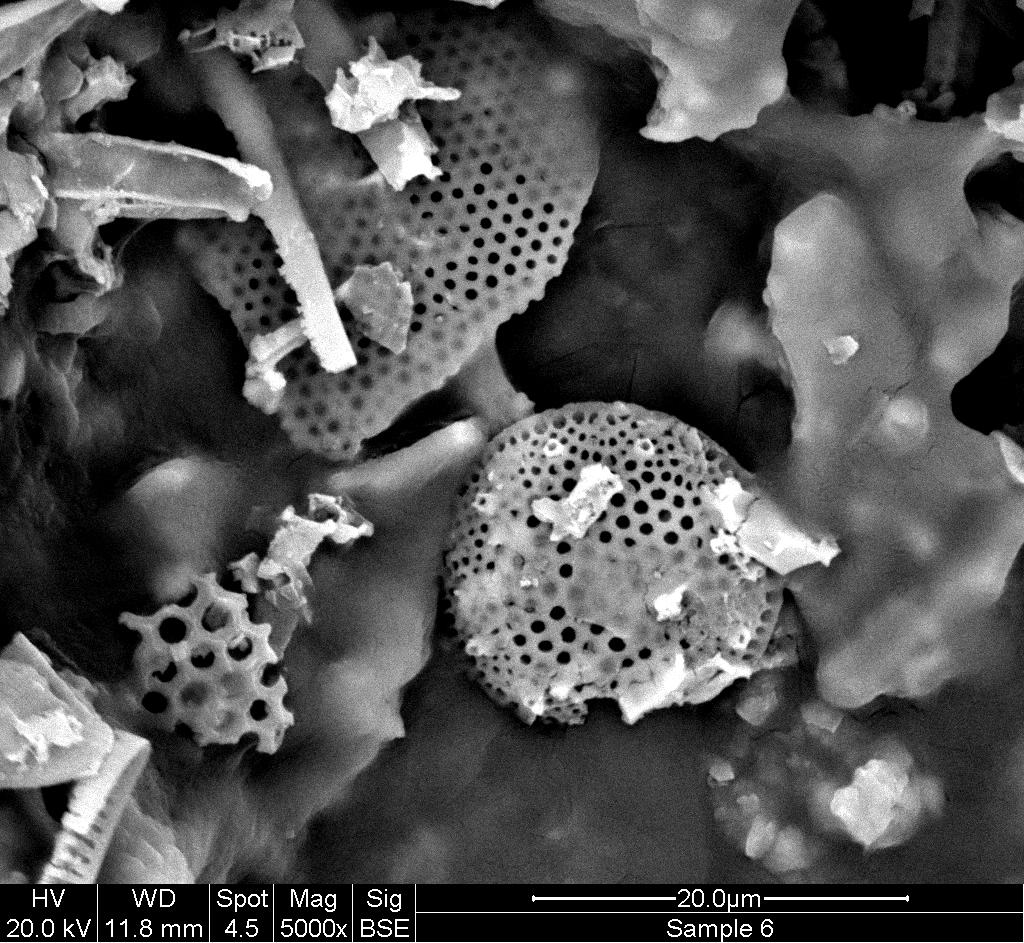 |
| C |  |
| 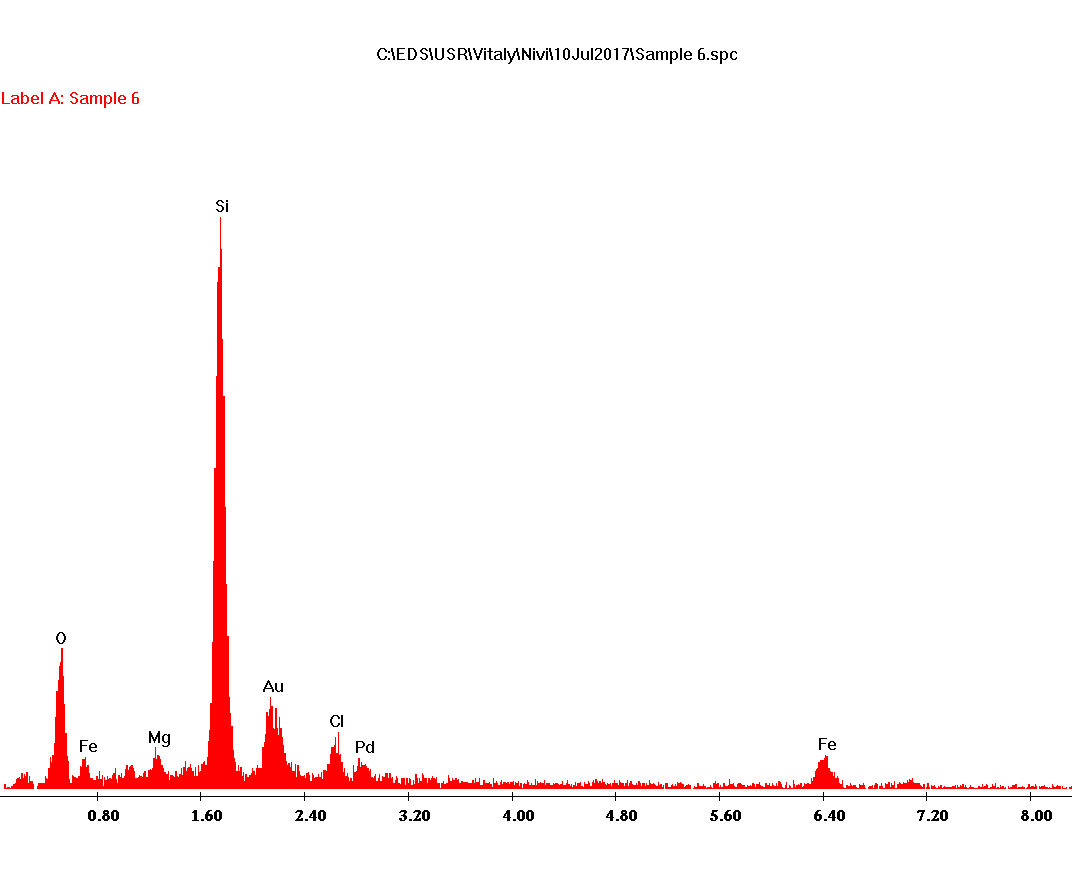  Fe-coating signal | |
| **Figure S2**: SEM micrographs of a colony incubated with Fe-coated diatom frustules for 24 hr (A). The colony trichomes are faintly visible in the background (cyan arrows). The colonies were not fixed prior to drying and did not maintain their natural morphology. This colony exhibited very strong particle collection, which resulted in the high particle load. Details of the area indicated by the red square (B) shows the morphology of the diatom frustules and the thin Fe-coating, which was detected by an EDS analysis (C). | |

| 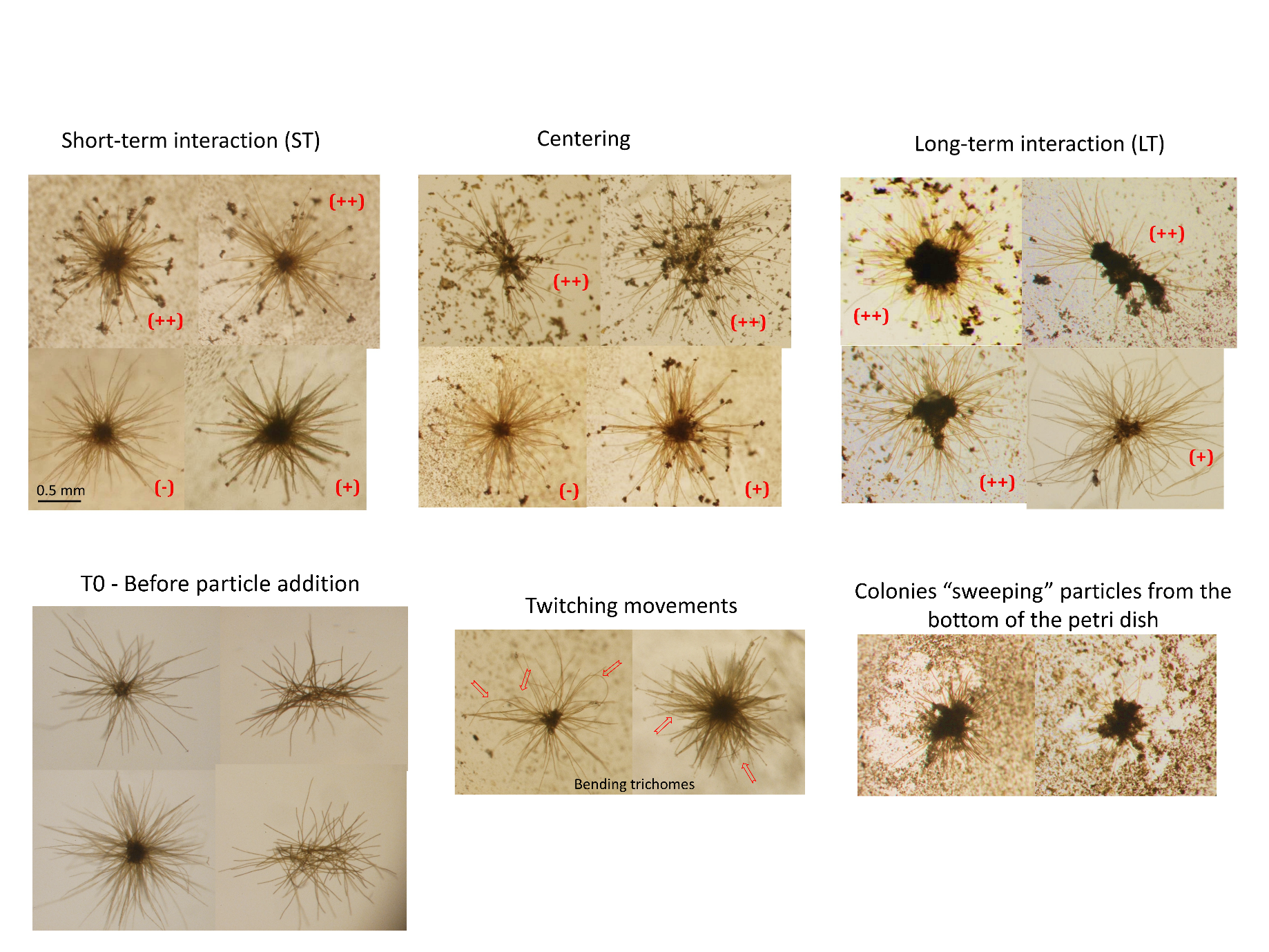 |
| --- |
| **Figure S3**: Microscopic observations of colony-dust interactions. **Top pictures**: exemplify the typical appearances of colonies that showed different degree of interaction, at the different stages. **Bottom-left**: typical appearance of colonies before particle addition. **Bottom-middle**: typical trichome bending (red arrows) in colonies that are actively collecting particles. **Bottom-right**: colonies at the end of the incubation (LT); in the background there are areas that have been cleaned of particles by intense particle collection. |

| A | B |
| --- | --- |
| 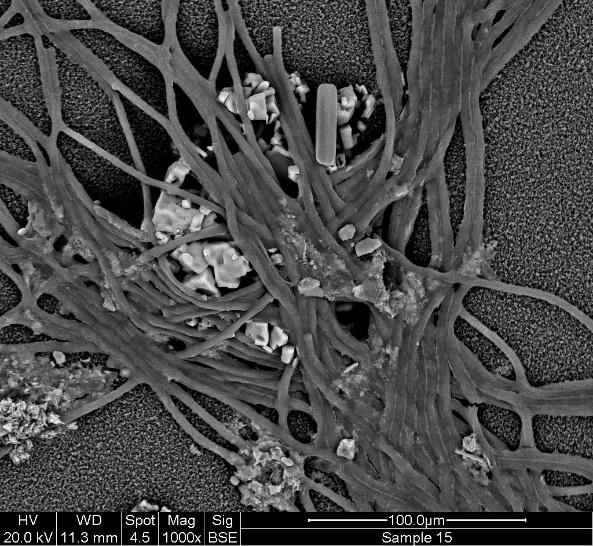 | 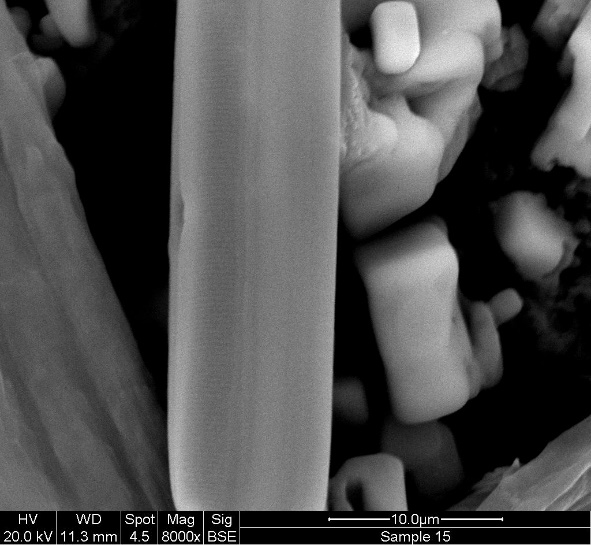 |
| C |  |
| 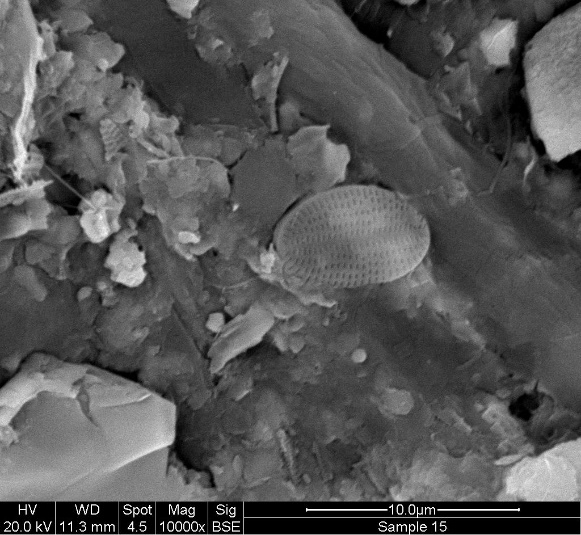 | **Figure S4**. (A) A SEM micrograph of a *Trichodesmium* puff-shaped colony, collected at the Gulf of Aqaba, in spring 2017. Enlarged areas (B,C) show diatom frustules (red arrows) that were associated to the colony. |

|  |
| --- |
| 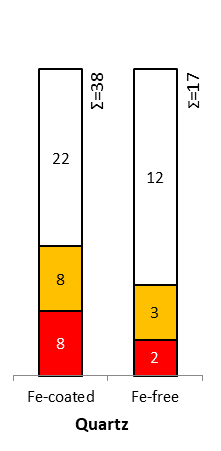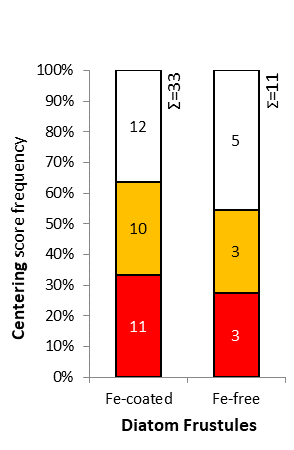 |
| **Figure S5: Accumulated centering scores of natural *Trichodesmium* colonies with silicate particles from all experiment days.**  The columns display the frequency of Centering scores for all colonies tested during the entire season. Interaction strength is noted by colors where red (++) = strong interaction, orange (+) = mild interaction, and white (-) = no interaction. The numbers of colonies in each category appear on the bars and the overall colony numbers at top of the bars. The difference between Fe-coated and Fe-free silicate particles was found to be statistically insignificant, using a 2- sided Fisher’s exact test (P-value threshold of 0.05). |

| A |
| --- |
| 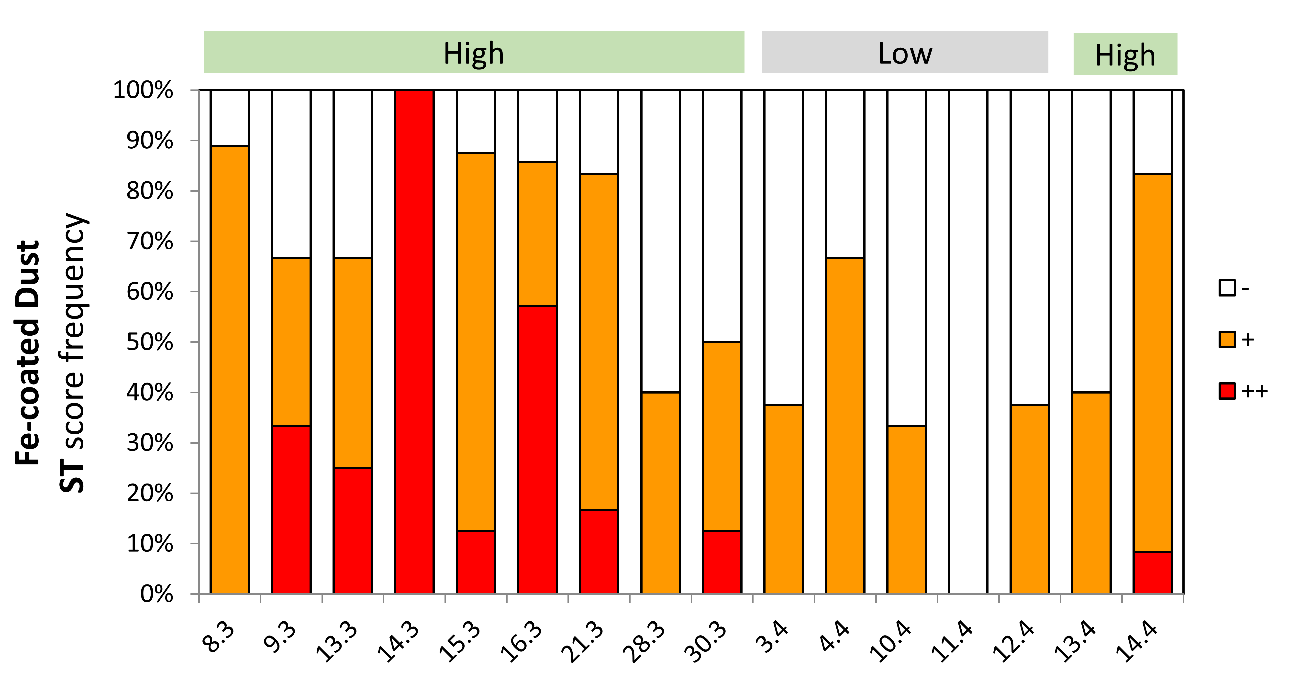 |
| B |
| 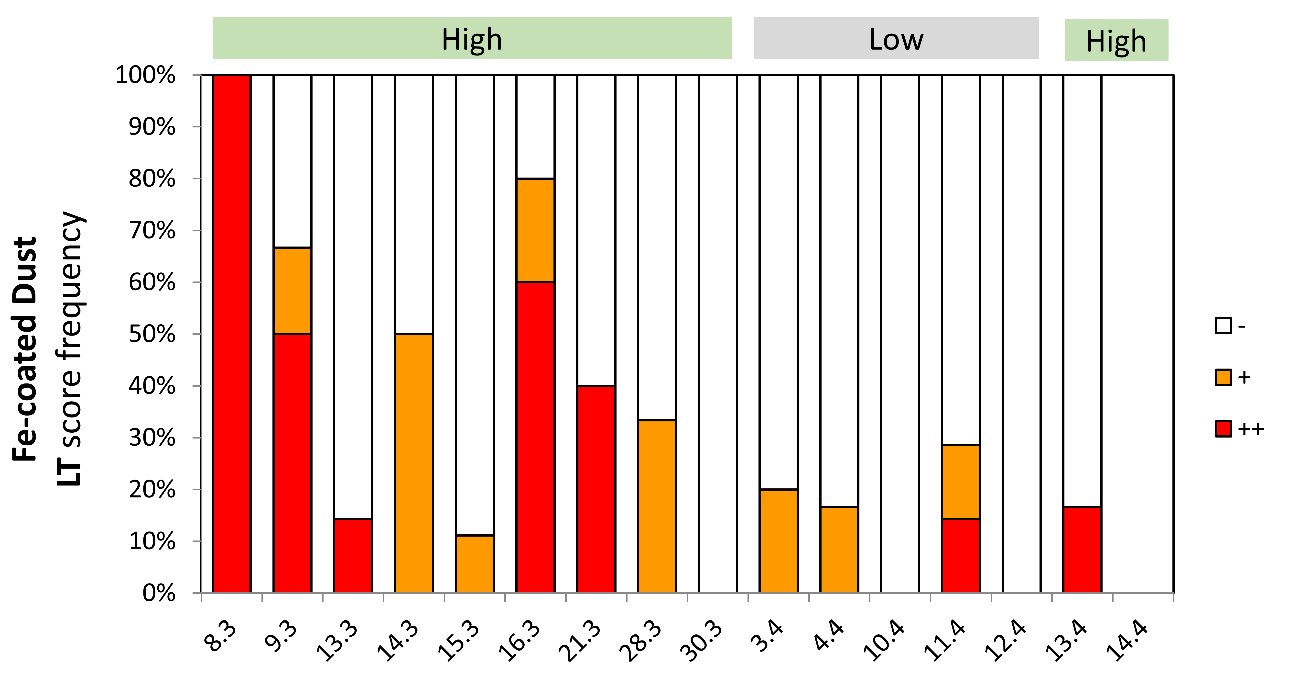 |
| **Figure S6. Temporal variations in the interaction of natural colonies with Fe-coated dust.** The columns display the frequency of ST (A) and LT (B) interaction scores, in each experiment day. The dates of the experiments are shown in X-axis labels. The green and grey horizontal bars indicate the periods referred to in section 3.3.3 as the ‘high period’ and ‘low period’, based on the level of ST interactions with dust. |

| 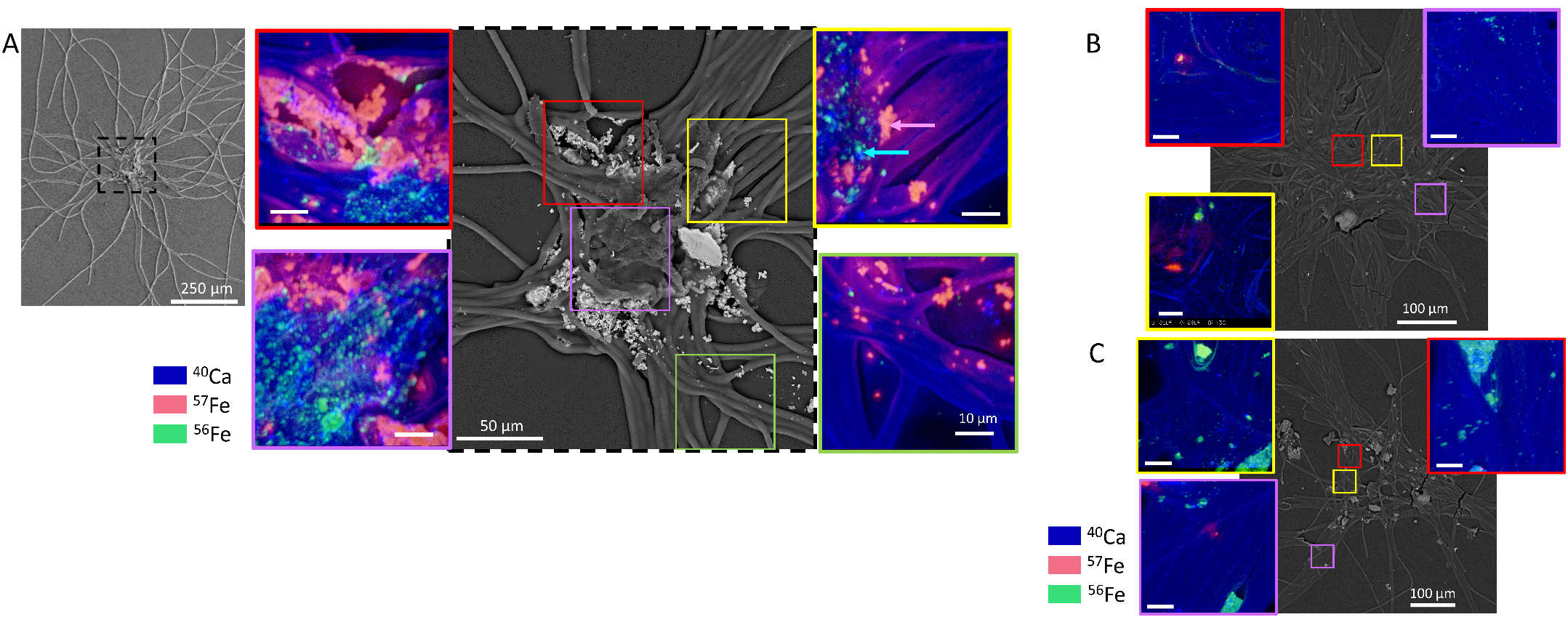 |
| --- |
| **Figure S7**: NanoSIMS images from (A) second colony (B-C) control colonies. Background image is a backscatter electron diffraction image, and inlaid images are 50 µm^2^ merged secondary ion images of surface distributions of ^57^Fe (pink), ^56^Fe (cyan) and ^40^Ca (blue). The distribution of the two Fe isotopes demonstrates the presence of the added ^57^Fe-Hematite particles alongside ambient ^56^Fe-rich particles. Ion image scale bar = 10 µm |

| 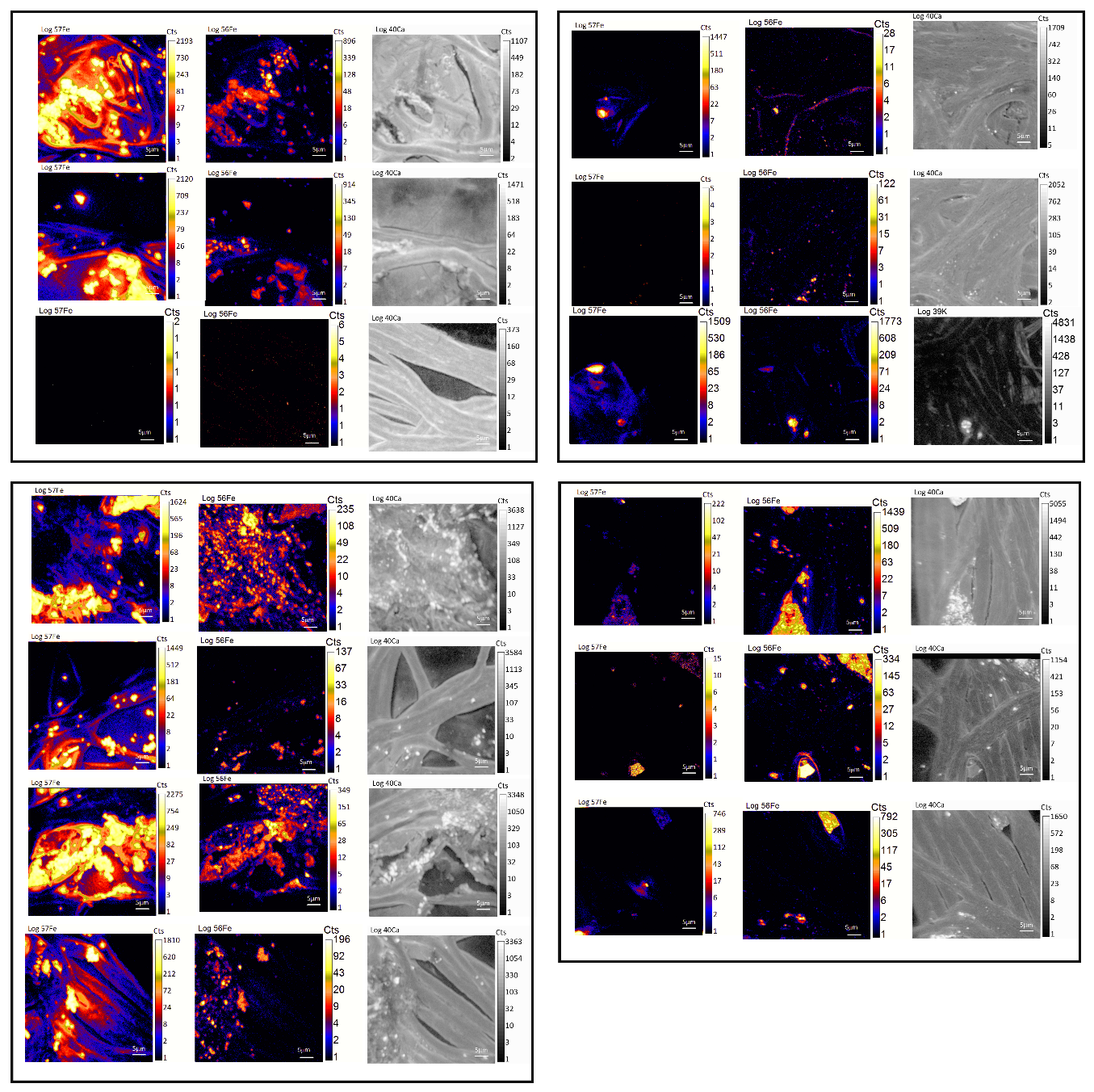 |
| --- |
| **Figure S8**: NanoSIMS single ion images from 3-4 locations on all four colonies analyzed. These ion images were used to generate overlay images. |

| A | **Figure S9: Rare associations between single IMS101 filaments and dust particles**. Single filament cultures were grown under Fe-deplete and Fe-replete conditions to mid-logarithmic and early-stationary growth stages. The cultures were then incubated with dust particles and inspected under a light microscope. In only 0.2%, of nearly 10,000 inspected filaments, we observed any association to particles (per treatment we observed a range of 0 to 1.4% positive interactions). In these rare positive cases, only one or two particles were identified (A-C: red circles). In some associations we observed transparent mucus connecting the filament and the particle (C: cyan arrows). The displayed trichomes (A-C) were grown for 10 days (mid-logarithmic phase) in YBCII media supplemented with 50 nM Fe; the pictures were taken after a 6 hr incubation with dust. Scale bars = 100 µm. |
| --- | --- |
| 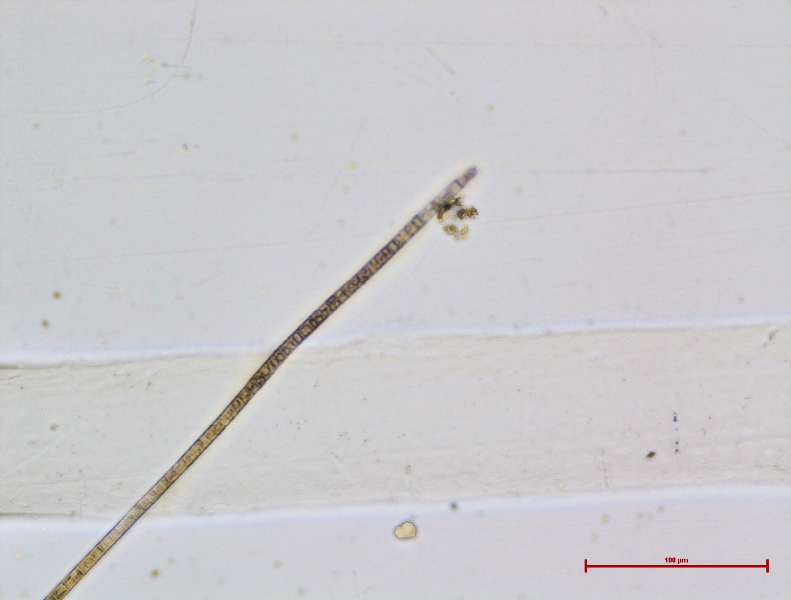 |  |
| B | C |
| 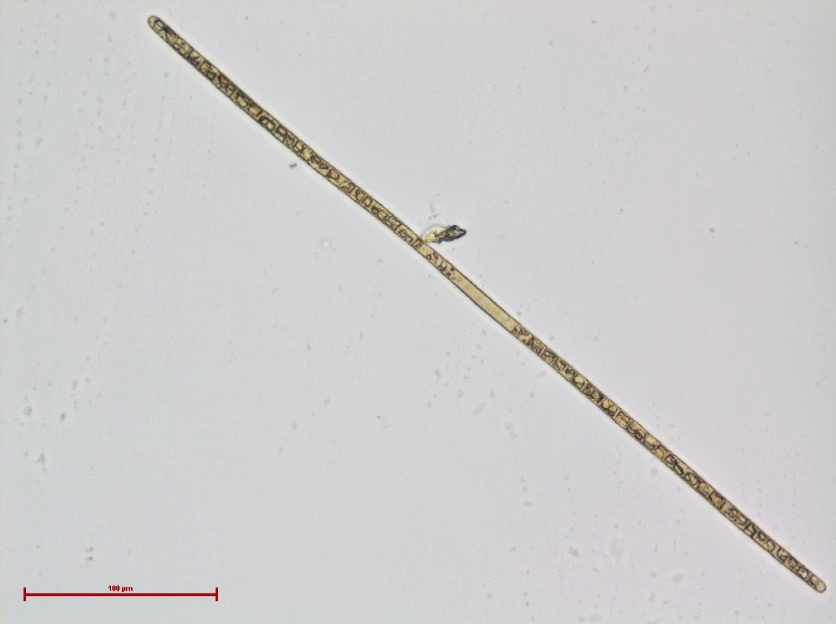 | 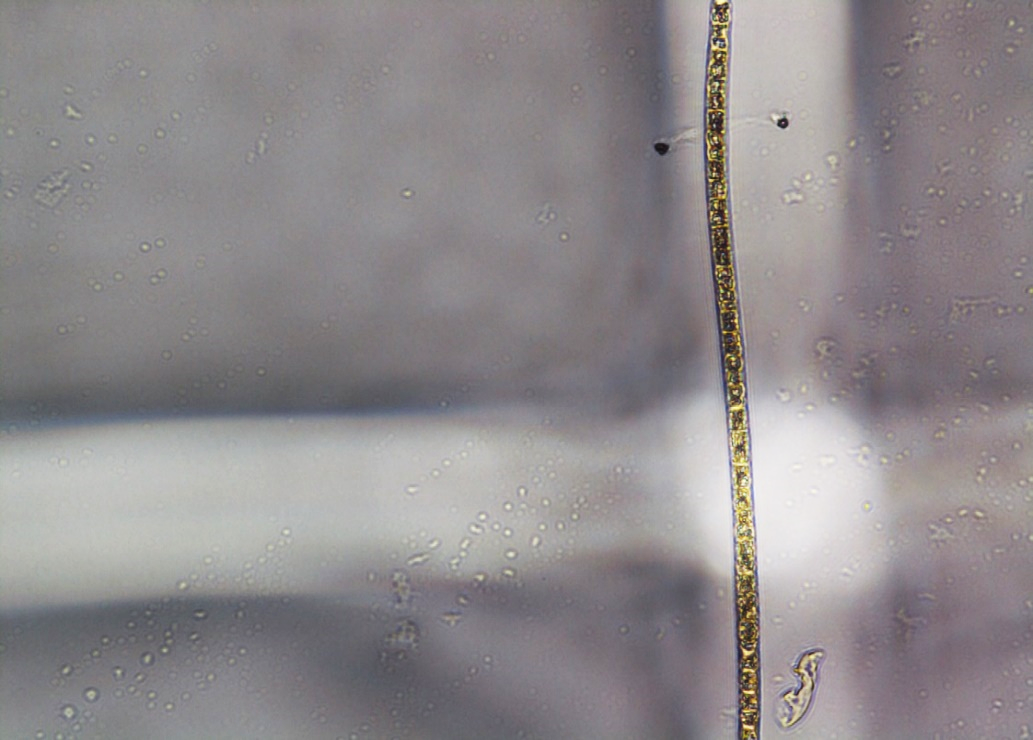 |

| A | B |
| --- | --- |
| 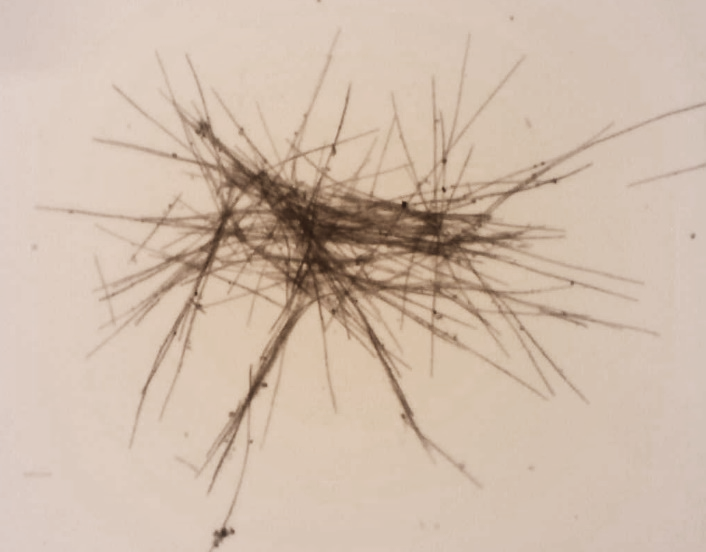 | 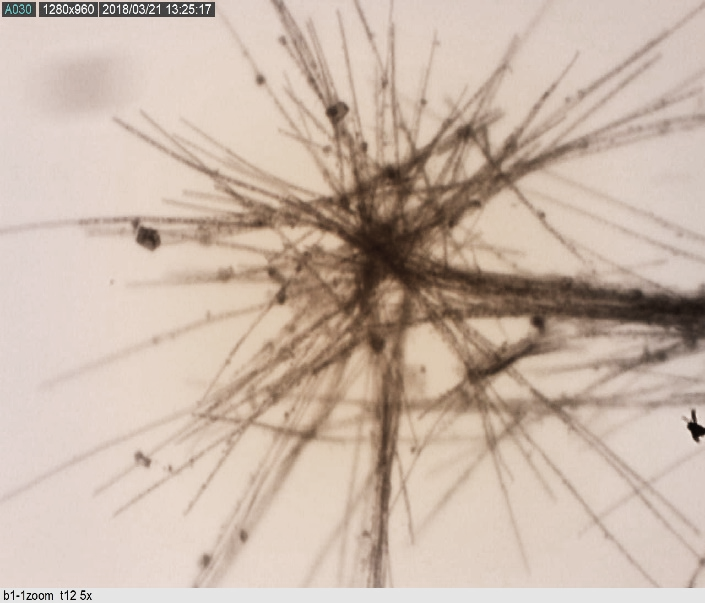 |
| **Figure S10: Association between IMS101 colonies and dust particles.** Cultures grown in Fe deplete conditions and tranferred to nutrient-poor FSW, formed abundant and stable colonies. The added dust adhered to all tested colonies (n=150). Tens to hundreds of particles adhered to each colony; a few of them are marked by the red arrows. The two displayed colonies were imaged after a 1 hr incubation with dust. Scale bar = 100 µm. | |

Table S1: Outline of particle incubations with natural *Trichodesmium* colonies.

|  | | **Particle type** | **Preparation** | **Experiment days (#)** | **Tested colonies per day** | **Total tested colonies** |
| --- | --- | --- | --- | --- | --- | --- |
| **Dust** | | Natural/untreated | Natural dust washed & suspended in DDW | 16 | 7-12 | 136 |
|  |  | Acid washed | Natural dust soaked in HCl (32%) for 10 days, washed & suspended in DDW | 16 | 7-12 | 136 |
|  |  | Fe-coated | Acid washed dust coated with Fe-oxides | 16 | 7-12 | 135 |
| **Silicate particles** | **Diatom frustules** | Fe-free | Diatomaceous Earth soaked in acid and washed in DDW | 8 | 5-10 | 57 |
|  |  | Fe-coated | Acid cleaned Diatomaceous Earth, coated with Fe-oxides | 8 | 5-9 | 56 |
|  | **Quartz** | Fe-free | Grinded mineral quartz minerals soaked in acid and washed in DDW | 6 | 6-9 | 48 |
|  |  | Fe-coated | Acid cleaned grinded mineral quartz, coated with Fe-oxides | 6 | 5-9 | 47 |

| Table S2: Statistical significance and strength of correlation. | | | | | | |
| --- | --- | --- | --- | --- | --- | --- |
| Strength of correlation (Cramer’s V)^b^ | | | P-value  (Fisher’s exact test) ^a^ | | | Compared treatment pairs |
| LT | Centering | ST | LT | Centering | ST |  |
| 0.3 | - | 0.2 | 0.002 | 0.2 | 0.009 | Dust / Fe-coated dust |
| 0.5 | 0.3 | 0.2 | 5e-10 | 2e-04 | 0.001 | Dust / Acid-washed dust |
| 0.2 | 0.2 | 0.2 | 0.009 | 0.006 | 0.04 | Fe-coated dust / Acid-washed dust |
| 0.4 | - | 0.4 | 0.001 | 0.6 | 2e-05 | Di / Fe-coated Di |
| 0.5 | - | 0.4 | 0.001 | 0.7 | 3e-04 | Qz / Fe-coated Qz |
| - | - | - | 0.6 | 0.4 | 0.06 | Di / Qz |
| - | - | - | 0.7 | 0.2 | 0.3 | Fe-coated Di / Fe-coated Qz |
| a - The P value threshold was set to 0.05 , all values above the threshold are colored red.  b - Presented only for pairs that are significantly different according to Fisher’s exact test. | | | | | | |

| **Table S3: Physical association between *Trichodesmium* culture, IMS101, and dust.** | | | | | | |
| --- | --- | --- | --- | --- | --- | --- |
| **Single filaments interaction with dust** | | | | | | |
| **Fe (nM)** | **Growth Phase** | **Density (Trichome/ml)** | **Total inspected trichomes** | | **Interaction (%)** | **repeats** |
| 50 | Mid-exponential | 290-1400 | 1800 | | 0-0.1 | 6 |
|  | Early-stationary | 240-490 | 3200 | | 0-0.8 | 6 |
| 400 | Mid-exponential | 270-690 | 1600 | | 0-1.4 | 6 |
|  | Early-stationary | 430-1500 | 3000 | | 0-1.1 | 6 |
| **Colony formation and interaction with dust** | | | | | | |
| **Culturing** | | **Experiment condition** | | | | |
| **Fe (nM)** | **Growth Phase** | **Media** | | **Density (Trichome/ml)** | **Colonies (colonies/ml)** | **Interaction (%)** |
| 50 | Mid-exponential | FSW | | 4400 | 60 | 100 |
|  | Early-stationary | FSW | | 6200 | 80 | 100 |
|  | Early-stationary | YBCII + 50 nM Fe | | 6200 | 0 | - |
| 100 | Early-stationary | FSW | | 8300 | <10 | - |
|  | Early-stationary | YBCII + 50 nM Fe | | 8300 | 0 | - |
